# Ventral hernia repair in high-risk patients and contaminated fields using a single mesh: proportional meta-analysis

**DOI:** 10.1007/s10029-022-02668-w

**Published:** 2022-09-13

**Authors:** S. Morales-Conde, P. Hernández-Granados, L. Tallón-Aguilar, M. Verdaguer-Tremolosa, M. López-Cano

**Affiliations:** 1 Unit of Innovation in Minimally Invasive Surgery, Department of General Surgery, University Hospital Virgen del Rocío, University of Sevilla, Seville, Spain; 2 General Surgery Unit, Fundación Alcorcón University Hospital, Rey Juan Carlos University, Alcorcón, Spain; 3 Abdominal Wall Surgery Unit, Department of General Surgery, Hospital Universitario Virgen del Rocío, c/ Asuncion 26, 2ºA, 41011 Seville, Spain; 4 Abdominal Wall Surgery Unit, Department of General Surgery, Hospital Universitari Vall d’Hebron, Universitat Autónoma de Barcelona, Barcelona, Spain

**Keywords:** Ventral hernia, Mesh, Biosynthetic mesh, Synthetic mesh, Biologic mesh

## Abstract

**Purpose:**

The use of mesh is a common practice in ventral hernia repair (VHR). Lack of consensus on which prosthetic material works better in different settings remains. This meta-analysis aims to summarize the available evidence on hernia recurrence and complications after repair with synthetic, biologic, or biosynthetic/bioabsorbable meshes in hernias grade 2–3 of the Ventral Hernia Working Group modified classification.

**Methods:**

A literature search was conducted in January 2021 using Web of Science (WoS), Scopus, and MEDLINE (via PubMed) databases. Randomized Controlled Trials (RCTs) and observational studies with adult patients undergoing VHR with either synthetic, biologic, or biosynthetic/bioabsorbable mesh were included. Outcomes were hernia recurrence, Surgical Site Occurrence (SSO), Surgical Site Infection (SSI), 30 days re-intervention, and infected mesh removal. Random-effects meta-analyses of pooled proportions were performed. Quality of the studies was assessed, and heterogeneity was explored through sensitivity analyses.

**Results:**

25 articles were eligible for inclusion. Mean age ranged from 47 to 64 years and participants’ follow-up ranged from 1 to 36 months. Biosynthetic/bioabsorbable mesh reported a 9% (95% CI 2–19%) rate of hernia recurrence, lower than synthetic and biologic meshes. Biosynthetic/bioabsorbable mesh repair also showed a lower incidence of SSI, with a 14% (95% CI 6–24%) rate, and there was no evidence of infected mesh removal. Rates of seroma were similar for the different materials.

**Conclusions:**

This meta-analysis did not show meaningful differences among materials. However, the best proportions towards lower recurrence and complication rates after grade 2–3 VHR were after using biosynthetic/slowly absorbable mesh reinforcement. These results should be taken with caution, as head-to-head comparative studies between biosynthetic and synthetic/biologic meshes are lacking. Although, biosynthetic/bioabsorbable materials could be considered an alternative to synthetic and biologic mesh reinforcement in these settings.

**Supplementary Information:**

The online version contains supplementary material available at 10.1007/s10029-022-02668-w.

## Introduction

Incisional ventral hernia repair is a common surgical practice. The use of mesh reinforcement has become a reference to prevent hernia recurrence [1, 2]. However, the variety of devices—with over 150 mesh products on the market—and surgical techniques make it difficult to evaluate prostheses performance [3, 4]. Lack of consensus remains regarding the best practices for ventral hernia repair (VHR) in the setting of a contaminated surgical field [2]. Permanent synthetic meshes are made of non-absorbable materials such as polyester, polypropylene, polyvinylidene fluoride, or polytetrafluoroethylene (PTFE). Although longer used, these prostheses are associated with a risk of postoperative infection and subsequent complications in contaminated or clean-contaminated settings [1, 5–7]. This previous context led to the development of biologic implants, as an alternative to synthetic meshes [7, 8]. However, concerns about their possible advantages remain [8–10].

Biosynthetic/slowly absorbable meshes have recently been introduced as a potential alternative to biological implants in contaminated abdominal wall reconstruction [6]. The absorption rates of these meshes may vary according to their composition: 6 months for polyglycolic acid (PGA): trimethylene carbonate materials (TMC), 12–18 months for poly-4-hydroxybutyrate (P4HB) component, and 12–36 months for a mixture of copolymer fibers of polyglycolide, polylactide, and polytrimethylene carbonate and fibers of polylactide and polytrimethylene carbonate, respectively, in comparison to classical glycolide and lactide mesh, which resorbs in less than two months [1, 11].

The Ventral Hernia Working Group (VHWG) 2010 classification system [5] stratifies patients with ventral hernia according to their risk for postoperative Surgical Site Occurrence (SSO), depending on the patient and wound characteristics, such as smoker, obese, diabetic, immunosuppressed, chronic obstructive pulmonary disease (COPD), or history of wound infection and hernia defects. Thus, it suggests the use of synthetic mesh, based on surgeon preference, for low-risk (grade 1) and the use of biologic mesh for increased risk and co-morbid patients (grade 2) as well as for contaminated or infected wounds (grades 3 and 4). However, the higher costs of biologic implants could have limited their use, even for high-risk patients [12]. In 2012 the VHWG classification was modified [13] stratifying patients as grade 1 (clean cases; low risk of complications), grade 2 (clean cases; co-morbidity, history of infection), or grade 3 (clean-contaminated, contaminated, and dirty wounds). In the setting of complex VHR, newly developed biosynthetic/slowly absorbable meshes have shown promising results for hernia recurrence and other postoperative complications [14].

In the previous context and given the lack of consensus on the most appropriate material for hernia repair in clean-contaminated and contaminated wounds, the present work aims to review the existing literature and gather evidence of postoperative outcomes of synthetic, biologic, and biosynthetic/slowly absorbable meshes in patients undergoing modified VHWG (2012) grade 2–3 complex ventral hernias.

## Methods

A systematic review and meta-analysis were conducted following the PRISMA (Preferred Reporting Items for Systematic Reviews and Meta-Analyses) 2020 statement [15] and the Meta-analysis of Observational Studies in Epidemiology guidelines [16]. The review was registered in the Prospective Register of Systematic Reviews (CRD42021265235).

### Search strategy

A literature search was conducted in January 2021. The search only considered those studies published from 2000 to 2021, written in English or Spanish, with the last search update on January 27. Search strategy was performed by an expert documentalist using the following databases: Web of Science (WoS), Scopus and MEDLINE (via PubMed). The search strategy included a combination of keywords and MeSH terms about hernia and mesh-related terms (see Supplementary file 1 for searching strategies in each database). Two independent reviewers (SMC and MLC) assessed the title and abstract of each record for inclusion and exclusion criteria. After preliminary screening, the selected publications were scanned in full text by the authors for eligibility. Discrepancies were solved after a discussion between reviewers.

### Study selection, data extraction and quality assessment

We established the inclusion criteria for study selection according to observational studies, as well as randomized control trials (RCTs) of any design, with adult patients (≥ 18 years of age) undergoing grade 2–3 complex VHR with prosthetic reinforcement, either with synthetic, biologic, or biosynthetic/slowly absorbable mesh. Hernia grade was defined according to the modified VHWG grading system [13]. Publications reporting on at least one of the following primary outcomes were included: hernia recurrence, SSO, as described by the VHWG including infection, wound dehiscence, seroma, or development of an enterocutaneous fistula [13] and Surgical Site Infection (SSI), according to the Center for Disease Control (CDC) [17] definition. Secondary outcomes of interest were 30 days re-intervention and infected mesh removal.

Exclusion criteria included no reported clinical outcomes of interest, preclinical studies and/or review articles, editorials, data from registries, articles with non-full text available or comments to a study, single case reports, studies related to parastomal, inguinal, or hiatal hernia repair, repair using either no mesh or two different meshes, as well as prophylactic mesh insertion. For duplicate data reported by the same author(s), the article with the longest follow-up period was selected [18, 19].

Data were extracted by two independent reviewers using a Microsoft®Excel (Microsoft® Corporation 2016) data extraction form. Predefined variables such as year of publication, study design, follow-up period and number of patients were retrieved. Results reported in the studies were recorded according to mesh type. In addition, patient characteristics and comorbidities associated with increased risk for postoperative infection were extracted, including age, sex, Diabetes Mellitus (DM), obesity (Body Mass Index > 30 kg/m^2^), Chronic Obstructive Pulmonary Disease (COPD), active smoker, and history of wound infection. Hernia characteristics and operative details were extracted as well: CDC wound classification [17], VHWG hernia grade [13], mesh type, setting of intervention (emergency or elective), repair technique and mesh placement location. In terms of postoperative complications, data on hernia recurrence, SSO, seroma, and SSI, as well as 30 days re-intervention, mesh removal and length of hospital stay were detailed. Those articles in which VHWG grade was not explicitly reported but had a cohort of patients with one or more of the comorbid conditions mentioned above, which allowed for a definition of VHWG grade, were selected and included in the review (classified as grade ≥ 2). Articles with grade 1–3 hernias for which outcomes were disaggregated by hernia grade were included in the analysis only collecting data for grade 2–3 outcomes. Discrepancies in data extraction were solved by the team of authors involved.

Bias of the included studies has been assessed using the Cochrane RoB 2 Tool for RCT [20] and robvis tool to create risk-of-bias plots [21]. The appropriate adaptations of the Newcastle–Ottawa scale were used for cohort or cross-sectional studies [22, 23]. This scale is based on a cumulative score in three categories: selection of study groups, comparability of their cases and controls and ascertainment of the outcome/exposure. There are two versions of the scale, one for cohort studies and one for case–control studies, and other version adapted from cross-sectional studies. If the assessed study includes criteria for an item, a score of one point is allocated to the selection and ascertainment of outcome/exposure category; two points can be allocated to the comparability category, resulting in a maximum of 9 points. We considered studies that received a score of 9–7 to have a low risk of bias, studies with 4–6 points as medium risk of bias, and studies with 0–3 points as a high risk of bias.

Two authors evaluated the articles’ methodological quality, and any discrepancies were solved through discussion with a third investigator.

### Data synthesis and statistical analysis

The meta-analyses were carried out considering one effect size per each study included, regardless of the type of mesh. A random-effects model was fitted to estimate pooled weighted proportions with corresponding 95% confidence intervals (CIs). CIs 95% excluding 0 were considered significant. Statistical heterogeneity was assessed using the χ^2^ and *I*^2^ statistics to analyse the extent of inconsistency and variation across the studies. Heterogeneity was considered high if *I*^2^ was > 50% [24]. Heterogeneity was explored with sensitivity analyses to reflect the influence of several subgroups on the pooled proportions adjusted by mesh type. The variables explored in the subgroup analysis were based on a time horizon of outcomes (studies with only long-term complications), population characteristics (studies with a patient with mean BMI ≥ 30 and mean BMI < 30, studies with a proportion of smokers ≥ 25% and smokers < 25%), study design (prospective vs retrospective) and risk of study bias (low vs moderate).

The possibility of publication bias was examined using the Doi plot and Luis Furuya-Kanamori asymmetry index (LFK index) [25] by a subgroup of mesh type. This approach has been suggested to be more robust for meta-analyses that include less than 10 studies. In the presence of symmetry, one can conclude as no publication bias but in the absence of symmetry, one can expect publication bias. This publication bias was measured by the asymmetry index (LFK index). An LFK index within ± 1, out of ± 1 but within ± 2, and >± 2 is to mean no asymmetry, minor asymmetry, and major asymmetry, respectively. The influence of potential publication bias on results was explored by using the trim-and-fill procedure [25]. *P* < 0.05 was considered statistically significant in all analyses. These were conducted using STATA (16.1, StataCorp LLC, College Station, TX).

## Results

### Study selection

**Fig. 1:**
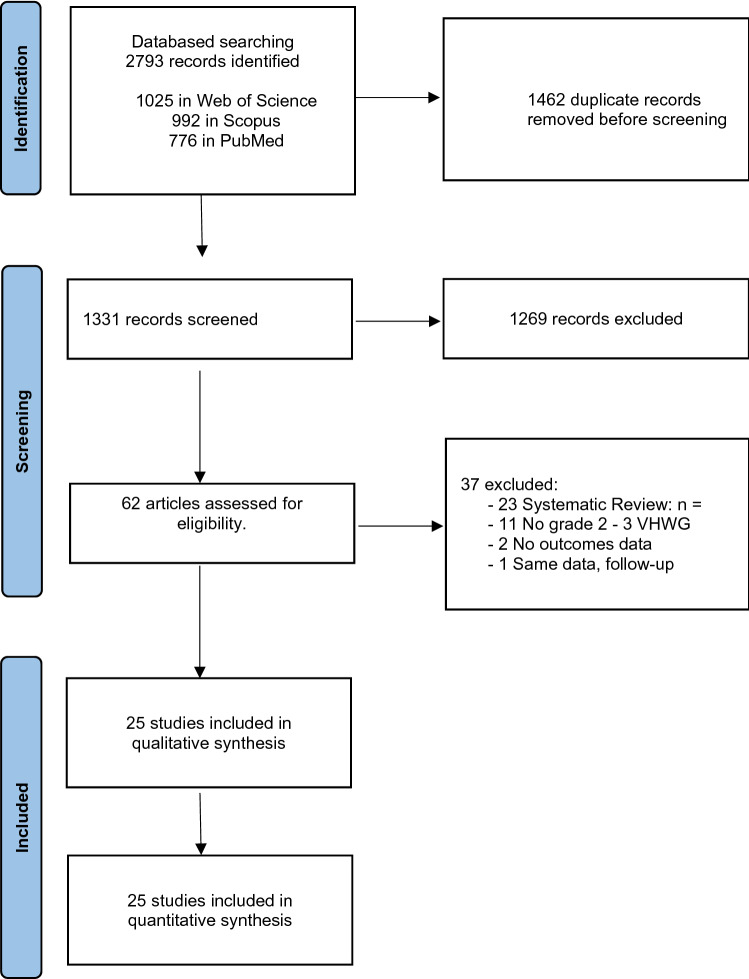
PRISMA flow diagram

A total of 2793 articles were identified in the primary literature search (Fig. 1). After the removal of duplicates, the literature search identified 1331 publications. Following the screening of the title and abstract, 62 articles were assessed for full-text review. 25 articles met the inclusion criteria and qualified for the study. The main reasons for exclusion after full-text assessment were existing systematic reviews, lack of information on hernia characteristics (VHWG grade 2–3), lack of information on patient comorbidities and contamination to define hernia grade if missing, and lack of results for outcomes of interest. A subsequent article with the same data from the same authors but with a longer follow-up replaced the publication with a shorter follow-up.

### Study characteristics

Of the 25 publications that met the inclusion criteria, only two were RCTs [8, 26]; 17 were observational retrospective studies [12, 14, 27–41] and six were observational prospective studies [1, 3, 6, 9, 19, 42]. Thirteen studies compared permanent synthetic to biologic mesh [8, 12, 26–32, 34–36, 38]; one publication compared synthetic to biosynthetic/slowly absorbable mesh [40]; two compared biosynthetic/slowly absorbable to biologic implant [14, 41] and, 9 articles presented outcomes of biosynthetic/slowly absorbable meshes [1, 3, 6, 9, 14, 19, 33, 39, 42] (six for P4HB mesh, two publications referred to PGA: TMC mesh, and one referred to both P4HB and PGA: TMC meshes. Among the studies using synthetic mesh, polypropylene was the most common material [8, 12, 26, 27, 29, 30, 32, 34, 35, 38, 40]. For biologic prosthetics, nine articles described porcine dermis meshes [8, 14, 26, 28, 32, 34, 35, 38, 41], whereas one article mentioned both porcine and human dermis prosthetics [29].

The 25 selected studies included a total of 3771 patients. Mean age ranged from 47 to 64 years and participants’ follow-up ranged from 1 to 36 months. A clear description of the VHWG hernia grade was reported in 13 studies [1, 3, 9, 26, 29–31, 33, 34, 37–40], although the remaining articles presented data on comorbid conditions which allowed for a definition of VHWG grade if patients had one or more of the listed comorbidities (hernias classified as grade ≥ 2) [6, 8, 12, 14, 27, 32, 35, 36, 41, 42]. Two articles reported on “high risk” patients, as those immunosuppressed and comorbid [19, 28]. In three studies with grade 1 hernias, operative outcomes were disaggregated by hernia grade, so only data on operative outcomes fulfilling the hernia grade criteria (grades 2 and 3) were derived separately [1, 33, 37]. Regarding wound contamination, most of the studies reported on CDC wound class [1, 6, 8, 9, 12, 19, 26, 27, 29, 30, 33–37, 39–42]. Surgical characteristics and outcomes are shown in Supplementary file 2. Only one article did not report any information on mesh location [27]. For the outcomes of interest, two studies lacked information on hernia recurrence [29, 40]; three articles did not present results on SSI [1, 28, 30]; 12 articles did not report on SSO [3, 14, 19, 26–32, 38, 41] while only five articles did not report on seroma [1, 14, 27, 30, 32]. Regarding operative technique, 12 studies reported on component separation techniques (CST) [1, 6, 9, 12, 29, 30, 33–38] (Table 1).

**Table 1:** Summary of study characteristics

References	Study design	Sample size (n)	Follow-up months (± SD or range)	VHWG grade	CDC	Mean age (± SD or range)	Type of mesh, *n*	Mesh material
Finch et al. [33]	Retrospective	56	21 (4–54)	I–III^a^ (2012)	1–3	63 (25–84)	Biosynthetic	PGA: TMC (Gore® Bio-A®)
Rosen et al. [6]	Prospective	104	24 ()	–	2–3	58 (27–91)	Biosynthetic	PGA: TMC (Gore® Bio-A®)
Messa et al. [37]	Retrospective	70	24 (12.2–41)	I–III^b^ (2012)	1–4	58.6 (23.2–81)	Biosynthetic	P4HB (Phasix™)
Pakula et al. [39]	Retrospective	20	21.1 (5–50) / 16.5 ()	II–III (2010)	1–3	47 (13)	Biosynthetic	P4HB (Phasix™)
Plymale et al. [42]	Prospective	31	24 ()	–	1–2	52 (44–62)	Biosynthetic	P4HB (Phasix™)
Rognoni et al. [3]	Prospective	75	26.4 (6.4)	II–III (2010)	–	59 (30–87)	Biosynthetic	P4HB (Phasix™)
Roth et al. [19]	Prospective	82	36 ()	*High-risk*	1	54.7 (12)	Biosynthetic	P4HB (Phasix™)
van Rooijen et al. [9]	Prospective	84	3 (2.8–3.3)	III (2010)	1–3	62.5 (12.4)	Biosynthetic	P4HB (Phasix™)
Vauclair et al. [1]	Prospective	29	12 ()	I–III^c^ (2012)	1–4	61 (13.3)	Biosynthetic	P4HB (Phasix™)
Sahoo et al. [40]	Retrospective	438	1 ()	II–III	2–3	64 (53–69)	Synthetic (380)	Polypropylene (macroporous)
			25.5 (14.5)			61 (51–70)	Biosynthetic (58)	P4HB (Phasix™), PGA: TMC (Gore Bio-A®)
Buell et al. [14]	Retrospective	73	–	–	–	52.5	Biosynthetic (31)	P4HB (Phasix™)
						56.9	Biologic (42)	Porcine (Strattice™)
de Vries et al. [30]	Retrospective	254	36 (0–153)	III (2012)	2–4	58 (13.6)	Synthetic (36)	Polypropylene (Vypro®, Ultrapro®, Physiomesh™ Proceed®, Dualmesh®, Prolene®)
							Biologic (69)	Porcine (Strattice™), Surgisis®
Harris et al. [8]	RCT	165	27.3 (15.6)	–	1–4	55.5 (11.1)	Synthetic (83)	Polypropylene (Ventralight™ ST)
			25.5 (14.5)			55.0 (11.5)	Biologic (82)	Porcine (Strattice™)
Renard et al. [41]	Retrospective	81	28.8 (15.7–47.6)	–	3–4	65 (56–76)	Absorbable (57)	Polyglactin (Vicryl®)
			27.7 (24.5–33.1)			63 (57–69)	Biologic (24)	Porcine (Strattice®)
Chamieh et al. [29]	Retrospective	58	7.9 ()	III–IV (2010)	2–4	–	Synthetic (24)	Polypropylene (lightweight and heavyweight)
			11.3 ()				Biologic (34)	Human dermis (FlexHD®), porcine submucosa-derived (Biodesign®), porcine dermis (XenMatrix ™, XCM®, Fortiva®, Strattice™)
Brescia et al. [28]	Retrospective	64	24 (7–36)	*High-risk*^d^	–	59.13 (4.4)	Synthetic (32)	Synthetic (NR)
						58.63 (4.8)	Biologic (32)	Porcine (Fortiva®)
Koscielny et al. [35]	Retrospective	187	27.3 (4.3)	–	1–3	60 (9.9)	Synthetic (24)	Polypropylene (Ultrapro®, Vypro®)
			23.5 (3.7)			58 (9.3)	Biologic (24)	Porcine submucosa-derived (Biodesign®)
Nockolds et al. [38]	Retrospective	23	17 (2–48)	III–IV (2010)	–	57 (20–76)	Synthetic (6)	Polypropylene (Ultrapro®, Proceed®)
							Biologic (17)	Porcine submucosa-derived (Biodesign®)
Bondre et al. [27]	Retrospective	761	15 (1–50)	–	1–4	49.4 (12.7)	Synthetic (303)	Polypropylene (lightweight and/or mid-weight)
							Biologic (167)	Non-cross-linked biologic
DeNoto et al. [31]	Retrospective	744	18 ()	III–IV (2010)	–	57.0 (12.4)	Synthetic (268)	Synthetic (NR)
						55.2 (12.1–12.6)	Biologic (177)	Acellular xenograft implant
Majumder et al. [12]	Retrospective	126	18.4 (9.6)	–	2–3	59.2 (12.3)	Synthetic (57)	Polypropylene (midweight, macroporous design)
			21.5 (10.5)			59.6 (11.7)	Biologic (69)	Porcine acellular dermal xenografts
Fischer et al. [34]	Retrospective	72	12.1 (1.6–38.6)	II (2010)	1	52.6 (12.0)	Synthetic (45)	Polypropylene (Marlex®)
						54.2 (11.2)	Biologic (27)	Acellular Dermal Matrix
Olavarria et al. [26]	RCT	87	12.4 (11.4–12.8)	II–IV (2010)	2–4	51 (12.1)	Synthetic (43)	Polypropylene (medium-density, macroporous)
						51 (9.9)	Biologic (44)	Porcine Acellular Dermal Matrix
El-Gazzaz et al. [32]	Retrospective	25	32.9 (38.2)	–	–	50.1 (13.1)	Synthetic (15)	Polytetrafluoroethylene, Polypropylene (NR)
						51.8 (12.7)	Biologic (10)	Porcine dermal collagen
López-Cano et al. [36]	Retrospective	62	22.2 (13.6)	–	2–3	62.8 (12)	Synthetic (48)	Non absorbable
			33.9 (20.8)				Biologic (14)	(NR)

*RCT* randomized clinical trial, *VHWG* Ventral Hernia Working group, *CDC* Center for Disease Control wound classification, *SD* Standard Deviation, *P4HB* poly-4-hydroxybutyrate, *PGA: TMC* polyglycolic acid: trimethylene carbonate, *NR* not reported

^a^Data on SSI is for 2–3 grade hernias

^b^Data disaggregated by hernia grade; grade 1 is not considered

^c^Outcome of interest, hernia recurrence, is only for grade 3

^d^High risk: those immunosuppressed and co-morbid (≥ grade 2) [28]

### Quality assessment

**Fig. 2:**
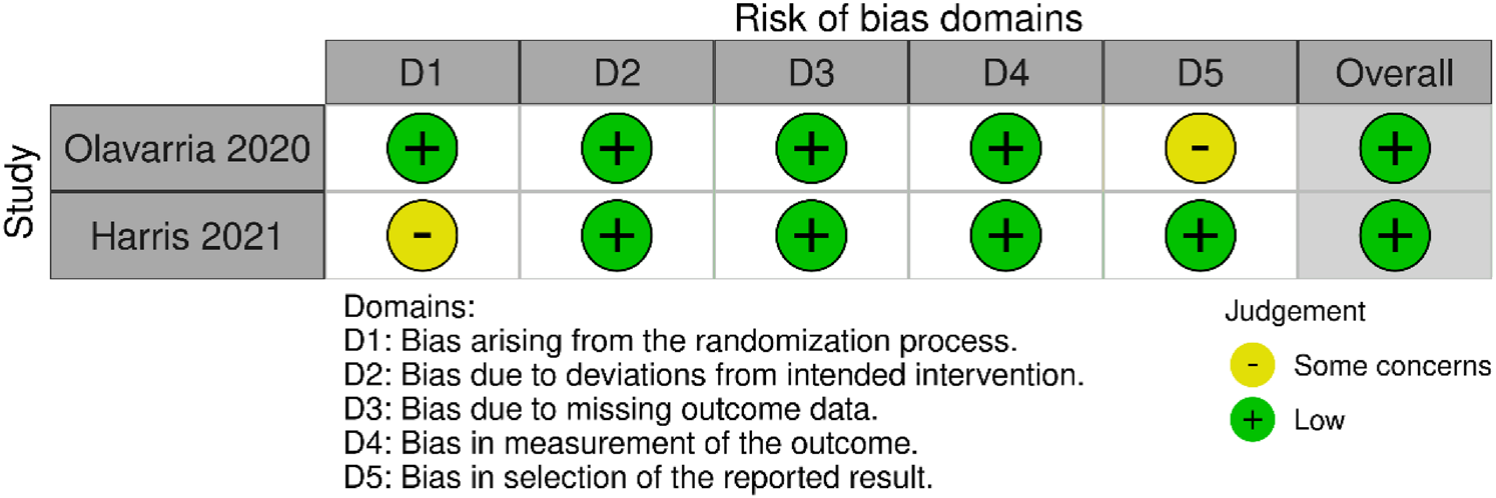
Assessment of risk for randomized controlled trials

The 2 RCT studies included in the review have an overall low risk of bias in the evaluation of methodological quality (Fig. 2). For the cohort studies, eleven obtained a high score and twelve a moderate score. The most frequent risks of bias were related to the risk of comparability based on the design or analysis (Table 2).

**Table 2:** Methodological quality evaluation for cohort/cross-sectional studies

Studies		Selection	Comparability	Results/Exposition	Total
Fischer	2014	****	**	***	9
Renard	2020	****	**	***	9
López-Cano	2017	****	*	***	8
Majumder	2016	****	*	***	8
Bondre	2016	***	**	***	8
Brescia	2016	****	**	***	8
Buell	2016	****	*	***	8
Sahoo	2017	****	*	**	7
de Vries	2020	***	*	***	7
Koscielny	2018	***	*	***	7
El-Gazzaz	2012	***	*	***	7
Finch	2021	***	–	***	6
DeNoto	2013	****	*	**	6
Rognoni	2020	***	–	***	6
Rosen	2017	***	–	***	6
Roth	2021	***	–	***	6
Messa et al	2019	***	–	***	6
van Rooijen	2020	***	–	***	6
Nockolds	2014	**	–	***	5
Pakula	2020	**	–	***	5
Plymale	2018	**	–	***	5
Vauclair	2021	**	–	***	5
Chamieh	2017	***	–	*	4

### Meta-analysis findings

#### Seroma

Rates of seroma are reported in 20 studies; however, only 13 articles reported in SSO rates. For this reason, only the former was included in the pooled analysis. 10 studies reported rates of seroma for biosynthetic/slowly absorbable meshes of 8% (95% CI 4–13%). A similar rate of 9% (95% CI 5–13%) was obtained from 11 studies on biologic mesh, as well as from the 11 articles on synthetic meshes, with a 9% rate of seroma (95% CI 5–13%). Heterogeneity ranged from *I*^2^ = 56.72% to *I*^2^ = 71.37% (Table 3).

**Table 3:** Summary of meta-analysis results according to type of mesh and clinical outcome

	No. studies	Sample size	Events	Pooled proportion (95% CI)	*I*^2^ (%)
*Seroma*					
Synthetic	11	804	66	9% (5–13%)	71.37
Biologic	11	544	44	9% (5–13%)	56.72
Biosynthetic	10	666	58	8% (4–13%)	73.09
*SSI*					
Synthetic	12	1090	214	18% (14–24%)	68.93
Biologic	13	731	178	27% (17–38%)	87.71
Biosynthetic	10	697	121	14% (6–24%)	89.91
*Infected Mesh*					
Synthetic	5	428	70	9% (0–25%)	92.33
Biologic	5	327	8	2% (0–7%)	64.60
Biosynthetic	4	222	1	0% (0–2%)	0
*Hernia Recurrence*					
Synthetic	12	940	151	13% (8–19%)	77.59
Biologic	14	792	165	20% (14–25%)	69.53
Biosynthetic	10	668	86	9% (2–19%)	93.32
*Re-intervention*					
Synthetic	5	559	45	6% (2–11%)	66.01
Biologic	7	348	43	11% (8–15%)	3.38
Biosynthetic	7	451	42	8% (5–12%)	37.40

*SSI* surgical site infection, *IC* confidence interval

#### Surgical site infection

**Fig. 3:**
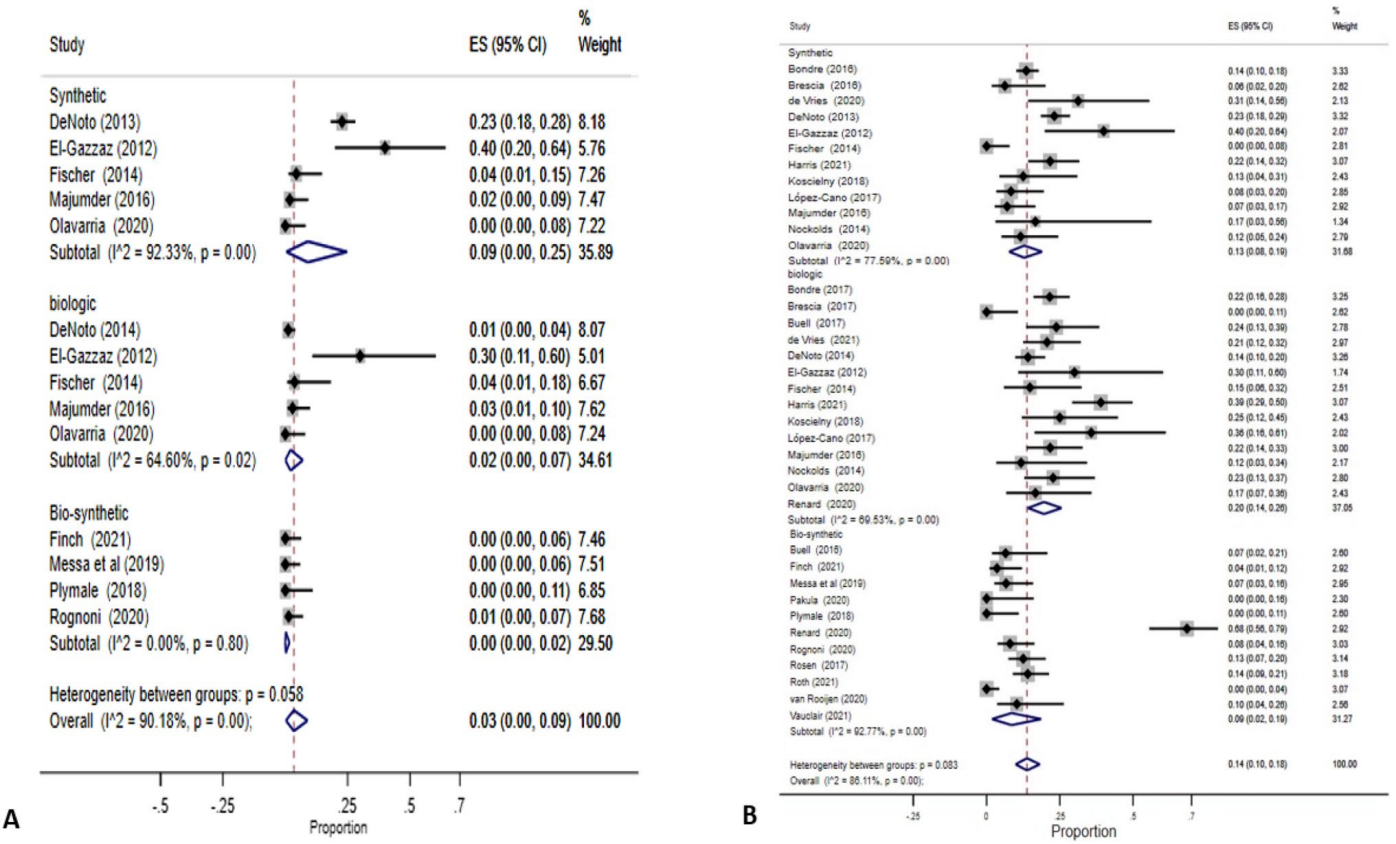
Estimation of infected mesh and hernia recurrence rates by mesh type. **a** Infected mesh rates by mesh type, **b** Hernia recurrence rates by mesh type

Ten studies reported on SSI rates for biosynthetic/slowly absorbable meshes. The pooled estimation showed lower SSI rates than biologic and synthetic mesh: 14% (95% CI 6–24%), compared to 18% (14–24%) for synthetic meshes (12 studies) and a higher rate of 27% (95% CI 17–38%) obtained from 13 articles reporting on biologic prothesis. There was high heterogeneity among studies (*I*^2^ statistics values between 68.93 and 89.91%; Fig. 3).

#### Infected mesh removal

Infected meshes were reported in 9 studies with a total of 79 events. The pooled estimation in the subgroup of biosynthetic/slowly absorbable mesh was 0% (95% CI 0–2%) with a very low heterogeneity (*I*^2^ = 0%) among 4 studies, compared with the subgroup of biologic and synthetic mesh, with pooled estimations of 2% (95% CI 0–7%) and 9% (95% CI 0–25%), respectively, and high heterogeneity among 5 studies (*I*^2^ = 64.60% and 92.33%).

#### Hernia recurrence

Meta-analysis showed that the rate of hernia recurrence was lower for biosynthetic/slowly absorbable mesh when compared to synthetic and biologic meshes. Ten studies on biosynthetic/slowly absorbable meshes reported an incidence of 9% (95% CI 2–19%), versus 12 studies on synthetic mesh, with a recurrence rate of 13% (95% CI 8–19%) and a hernia recurrence of 20% (95% CI 14–25%) obtained for the 14 studies on a biologic mesh. The *I*^2^ statistic reported substantial heterogeneity among the studies (93.32%, 77.59% and 69.53% for biosynthetic/bioabsorbable, synthetic, and biologic mesh, respectively).

#### Re-intervention

30 days re-invention was reported in 5 studies for synthetic mesh and 7 studies for biologic and biosynthetic/slowly absorbable each. Meta-analysis resulted in a pooled re-intervention estimation of 8% (5–12%) for biosynthetic/slowly absorbable and 11% (95% CI 8–12%) for biologic. Heterogeneity was low-moderate *I*^2^ = 37.40% and *I*^2^ = 3.38%.

### Sensitivity analysis

For the sensitivity analysis, we first analysed the subgroup of studies reporting results on long-term complications. In this sense, only 3 studies [9, 29, 40] reported short-term complication outcomes of seroma, SSI, and reintervention derived from the use of biosynthetic and synthetic meshes. The two studies with short-term complications of synthetic mesh [29, 40] had a seroma rate of 0% (95% CI 0–14%) [29] and 4% (95% CI 2–8%) [40], SSI rate of 21% (95% CI 9–40%) [29] and 11% (95% CI 7–16%) [40], and reoperation rate of 0% (95% CI 0–14%) [29] and 4% (95% CI 2–18%) [40]. Similarly, studies reporting short-term complications of biosynthetic mesh reported a seroma rate of 2% (95% CI 0–9%) [40] and 8% (95% CI 4–16%) [9], SSI of 22% (95% CI 14–35%) [40] and 13% (95% CI 7–22%) [9] and reoperation rate of 14% (95% CI 7–25%) [40]. After removing these 3 articles, we pooled subgroup analysis only for studies with long-term complications of meshes. Results are shown in Supplementary file 4. We can observe a few differences with respect to the results of the total pooled sample by mesh type. Seroma and SSI rates increase slightly in synthetic and biosynthetic mesh [from 9 to 11% (95% CI 6–17%] and from [18 to 20% (95% CI 15–25%)], for the respective long-term complications. In contrast, the long-term reintervention rates for synthetic mesh increase from 6 to 10% (95% CI 7–13%) and remain unchanged for biosynthetic mesh.

To explore risk factors, two subgroups were analysed, considering the main patient characteristics influencing the outcomes of interest. Results are shown in Supplementary file 4. In the subgroup of studies whose patients had an average BMI ≥ 30, there is a higher rate of hernia recurrence, infected mesh, and SSI for biologic mesh. However, complication rates are lower for synthetic mesh, apart from seroma. For biosynthetic/slowly absorbable mesh, there is a trend towards lower rates of SSI, infected mesh removal and hernia recurrence, although this percentage remains higher for the re-intervention variable. The subgroup of studies with, at least, 25% of smokers, presented higher rates of seroma, infected mesh, and re-intervention, independently of mesh type, although lower rates are observed for hernia recurrence if compared to the subgroup smoker < 25%. For the subgroups analysed (BMI ≥ 30, smoker ≥ 25%) mesh type does not seem to influence the outcomes. These analyses present significant heterogeneity and wide confidence intervals, which shows that differences among complication rates in patient subgroups are not significant. Similar results are obtained in sensitivity analyses for study design and risk of bias. There were only eight studies with a prospective design: six out of them were single-arm studies assessing results on biosynthetic/slowly absorbable mesh use and, the remaining were RCTs that compared biologic to synthetic mesh. Outcomes of biosynthetic/slowly absorbable mesh in the prospective studies, which represented a larger sample of studies, although not significant, show a lower rate for seroma, SSI, and recurrence. Finally, the subgroup analysis that classified studies according to low risk and medium risk of bias showed that studies with lower risk had a higher rate of SSI, hernia recurrence and re-intervention for biosynthetic/slowly absorbable and biologic mesh. Again, these results are not significant.

#### Publication bias

There was evidence of major publication bias regarding the proportion of seroma for each type of mesh, SSI for biologic, infected mesh for synthetic and biologic as indicated by the LFK index. The obtained LFK index and Doi plot based on the outcome by a subgroup of mesh type is presented in Supplementary file 3. The ‘‘trim and fill’’ procedure did not find any possible “missing” study.

## Discussion

Based on meta-analysis findings, the rate of hernia recurrence seems lower after biosynthetic/slowly absorbable mesh, with a pooled proportion of 9% recurrence as opposed to synthetic and biologic repair, with a recurrence of 13% and 20%, respectively. Mellia et al. [43] reviewed clinical outcomes of P4HB biosynthetic mesh and reported a similar recurrence of 9.1%. By contrast, the study by Renard et al. [41] compared the efficacy of rapidly absorbable polyglactin mesh versus biologic implant and reported a 68% recurrence for the former. This variability among the results might come from grouping different prosthetic materials within the same mesh category, as their behaviour and health outcomes may differ. Hodgkinson et al. [44] also stratified their analysis by repair method, although they included suture repair alone without mesh placement. Reported hernia recurrence was higher in this study as grade 2 hernias were ruled out: recurrence rate was 21% for non-absorbable synthetic mesh and 28.5% for biologic materials. Pooled estimation for SSI also showed a trend towards lower rates for biosynthetic/slowly absorbable mesh repair, with a 14% rate, compared to 18% for synthetic meshes and a higher rate of 27% for biologic repair. In the same systematic review, Mellia et al. [43] reported a 6.8% rate for SSI. This difference might come from the percentage of grade 1 hernias included in their analysis, associated with a lower risk of postoperative complications and recurrence, with no comorbidities or previously infected wounds. They suggest that anti-microbial properties of P4HB for the repair of contaminated wounds might explain the lower incidence of SSI in their study. In addition, available data for seroma revealed a rate of 8% for biosynthetic/slowly absorbable mesh, similar to the 9% reported for synthetic and biologic materials. In the present analysis, no infected meshes were removed after repair with biosynthetic/slowly absorbable mesh, with very low heterogeneity (*I*^2^ = 0%) among the studies, whereas rates of 2% and 9% were estimated for biologic and synthetic meshes, respectively, with high heterogeneity. However, this result should be interpreted with caution, given the small number of studies reporting on the infected mesh. Re-intervention resulted in an 8% rate for biosynthetic/slowly absorbable mesh and 11% for biologics.

This analysis, however, presents several limitations. Data have been reported as a percentage of complications (in absolute terms) derived from each mesh, rather than relative odds ratios from studies comparing one mesh versus another. The reason is the lack of comparative studies of biosynthetic/slowly absorbable mesh with other materials (most of them are single-arm studies). Only two RCTs [8, 26] were included in the review, although their quality assessment reported a low risk of bias. Most of the retrieved publications were retrospective observational studies with a moderate risk of bias because of a lack of comparability of the cases, their design, or the type of analysis. In addition, the *I*^2^ statistic reported substantial heterogeneity among the studies for the outcomes of interest. Sensitivity analyses performed to assess heterogeneity have shown that patient characteristics are not a source of this heterogeneity. Similar results are reported in the review by Samson et al. [45]. This review included studies using biological implants in open VHR and analyzed reporting data on early complications, late complications, and recurrences. Thus, reasons for the heterogeneity might come from other demographic characteristics of the patients, different methodologies, repair strategies, and prosthetic materials used in the studies, which limits the comparison of variables between the articles and makes it difficult to derive sound conclusions. However, as other authors stated, we must bear in mind that “…in proportional meta-analysis, *I*^2^ is usually high. This can be due to the nature of proportional data, where little variance is observed even in studies with small sample size. Moreover, true heterogeneity is expected in prevalence and incidence estimates due to differences in the time and place where included studies were conducted. Therefore, high I^2^ in the context of proportional meta-analysis does not necessarily mean that data is inconsistent. As such, the results of this test should be interpreted conservatively” [46].

Moreover, a clear description of the VHWG hernia grade was only reported in 13 studies [1, 3, 9, 26, 29–31, 33, 34, 37–40]. Thus, the grade had to be derived by the reviewers from the patient and wound characteristics. In our search of literature, we sought to select comparative studies for synthetic meshes and biologic implants. On the other hand, for biosynthetic/slowly absorbable meshes, given the lack of comparative publications, we allowed for the inclusion of single-arm studies. The inclusion of these articles could lead to a higher heterogeneity in the results. In addition, there is no universal acceptance of the VHWG grading scale [13].

Also, a recent review includes 274 studies with 28,868 patients to describe till 63 individual predictors for hernia recurrence [47], but we have only considered in our methodology 5 selected items (seroma formation, wound infection, mesh removal, hernia recurrence and re-intervention), in our opinion most important and appropriate to reach our goal.

Finally, our study does not include an analysis of data on preclinical testing and animal studies, which could supplement the scientific evidence for safety and effectiveness.

In conclusion, meta-analysis did not show meaningful differences among materials; although, it seems to be a trend towards lower recurrence and complication rates after grade 2–3 VHR using biosynthetic/slowly absorbable mesh reinforcement. Therefore, biosynthetic/slowly absorbable mesh could be an interesting option as an alternative to synthetic meshes and biologic implants in 2–3 grades. Also, these results should be interpreted with caution because of the lack of direct comparisons between biosynthetic/slowly absorbable versus synthetic meshes and/or biological implants. Differences in patient characteristics and the selection bias in single-arm observational studies make it difficult to have conclusive results.

Future research should focus on randomized controlled trials with defined control groups to allow for head-to-head comparisons among the different mesh materials like *COMpACT-BIO* study (RCT NCT04597840, clinicaltrials.gov) whose purpose is to investigate the clinical and economic benefit of the use of biosynthetic mesh in contaminated incisional hernia repair in comparison to the standard of repair. This is a multicenter, prospective longitudinal and randomized study, which also offers a standardized technique of repair, that still is open and in a recruitment phase.

Finally, due to the cost differences between the different types of materials, this information should be supplemented with registers, cost-effectiveness studies and/ or budget impact analyses informing about the health care value of the three alternatives.

## Supplementary Information

Below is the link to the electronic supplementary material.

Supplementary file1 (DOCX 209 KB)
