# Correction: Ventral hernia repair in high-risk patients and contaminated fields using a single mesh: proportional meta-analysis

**DOI:** 10.1007/s10029-022-02705-8

**Published:** 2022-11-01

**Authors:** S. Morales-Conde, P. Hernández-Granados, L. Tallón-Aguilar, M. Verdaguer-Tremolosa, M. López-Cano

**Affiliations:** 1 Unit of Innovation in Minimally Invasive Surgery, Department of General Surgery, University Hospital Virgen del Rocío, University of Sevilla, Seville, Spain; 2 General Surgery Unit, Fundación Alcorcón University Hospital, Rey Juan Carlos University, Alcorcón, Spain; 3 Abdominal Wall Surgery Unit, Department of General Surgery, Hospital Universitario Virgen del Rocío, c/ Asuncion 26, 2ºA, 41011 Seville, Spain; 4 Abdominal Wall Surgery Unit, Department of General Surgery, Hospital Universitari Vall d’Hebron, Universitat Autónoma de Barcelona, Barcelona, Spain

## Correction: Hernia 10.1007/s10029-022-02668-w

The article Ventral hernia repair in high-risk patients and contaminated fields using a single mesh: proportional meta-analysis, written by S. Morales-Conde, P. Hernández-Granados, L. Tallón-Aguilar, M. Verdaguer-Tremolosa and M. López-Cano, was originally published electronically in SpringerLink on 13 September 2022 without open access. After publication online with the author(s)’ decision to opt for Open Choice the copyright of the article changed on 27 October 2022 to © The Author(s) 2022 and article is licensed under a Creative Commons Attribution 4.0 International License, which permits use, sharing, adaptation, distribution and reproduction in any medium or format, as long as you give appropriate credit to the original author(s) and the source, provide a link to the Creative Commons licence, and indicate if changes were made. The images or other third party material in this article are included in the article's Creative Commons licence, unless indicated otherwise in a credit line to the material. If material is not included in the article's Creative Commons licence and your intended use is not permitted by statutory regulation or exceeds the permitted use, you will need to obtain permission directly from the copyright holder. To view a copy of this licence, visit http://creativecommons.org/licenses/by/4.0/.

The original article has been corrected.

