# Supplementary material for: Ventral hernia repair in high-risk patients and contaminated fields using a single mesh: proportional meta-analysis
Source: Hernia. 2022 Sep 13;26(6):1459–71. doi: 10.1007/s10029-022-02668-w (PMC9684228; doi:10.1007/s10029-022-02668-w)
Supplement: Supplementary file 1 — Supplementary file1 (DOCX 209 KB) [file 10029_2022_2668_MOESM1_ESM.docx]

**Supplementary file 1**

**Database and search strategies**

**The following databases were searched on 27 January 2021**

Web of Science, n: 1025

Scopus, n: 992

Pubmed, n: 776

Total= 2793 duplicates removed=1462, Records screened= 1331

Pubmed- Search strategy

**((("Hernia, Ventral"[Mesh] OR "Ventral Hernia"[Title/Abstract] OR "Ventral Hernias"[Title/Abstract] OR "Hernia, Umbilical"[Mesh] OR Exomphalo*[Title/Abstract] OR "Umbilical Hernia"[Title/Abstract] OR "Umbilical Hernias"[Title/Abstract] OR "Incisional Hernia"[Mesh] OR "Incisional Hernia"[Title/Abstract] OR "Incisional Hernias"[Title/Abstract] OR "Postoperative Hernia"[Title/Abstract] OR "Postoperative Hernias"[Title/Abstract] OR "midline hernia"[Title/Abstract] OR "lateral hernia"[Title/Abstract] OR "midline hernias"[Title/Abstract] OR "lateral hernias"[Title/Abstract]))**

OR

**((Ventral*[Title/Abstract] OR umbilical*[Title/Abstract] OR incisional*[Title/Abstract] OR epigastric*[Title/Abstract] OR Spigelian*[Title/Abstract]) AND (hernia*[Title/Abstract])))**

AND

**((("Biosynthetic Mesh"[Title/Abstract] OR "Biosynthetic Meshes"[Title/Abstract] OR "biological Meshes"[Title/Abstract] OR "biological Mesh" [Title/Abstract] OR "synthetic mesh"[Title/Abstract] OR "synthetic meshes"[Title/Abstract] OR "GORE BIO-A"[Title/Abstract] OR "TIGR Matrix"[Title/Abstract] OR "Phasix mesh"[Title/Abstract]))**

OR

**((Biosynthetic*[Title/Abstract] OR biological*[Title/Abstract] OR synthetic*[Title/Abstract] OR biologic*[Title/Abstract] OR "Phasix" [Title/Abstract] OR "Bio-a" [Title/Abstract] OR Tigr[Title/Abstract] OR Seri[Title/Abstract] OR "Reinforcement prosthesis"[Title/Abstract] OR polyglycolic[Title/Abstract] OR "trimethylene carbonate" [Title/Abstract] OR polylactide[Title/Abstract] OR "poly-4-hydroxybutyrate" [Title/Abstract] OR P4HB[Title/Abstract] OR "reabsorbable"[title/abstract] OR absorbable[title/abstract]) AND (Mesh*[Title/Abstract])))**

**Web of Science- Search strategy**

((("Ventral Hernia" OR "Ventral Hernias" OR Exomphalo* OR "Umbilical Hernia" OR "Umbilical Hernias" OR "Incisional Hernia" OR "Incisional Hernias" OR "Postoperative Hernia" OR "Postoperative Hernias" OR "midline hernia" OR "lateral hernia" OR "midline hernias" OR "lateral hernias"))

OR
((Ventral* OR umbilical* OR incisional* OR epigastric* OR Spigelian*) AND (hernia*)))

AND

((("Biosynthetic Mesh" OR "Biosynthetic Meshes" OR "biological Meshes" OR "biological Mesh" OR "synthetic mesh" OR "synthetic meshes" OR "GORE BIO-A" OR "TIGR Matrix" OR "Phasix mesh"))

OR

((Biosynthetic* OR biological* OR synthetic* OR biologic* OR Phasix OR Bio-a OR Tigr OR Seri OR "Reinforcement prosthesis" OR polyglycolic OR "trimethylene carbonate" OR polylactide OR poly-4-hydroxybutyrate OR P4HB OR reabsorbable OR absorbable) AND (Mesh*)))

Scopus- Search strategy

TITLE-ABS-KEY("Ventral Hernia") OR TITLE-ABS-KEY("Ventral Hernias") OR TITLE-ABS-KEY("Exomphalos") OR TITLE-ABS-KEY("Umbilical Hernia") OR TITLE-ABS-KEY("Umbilical Hernias") OR TITLE-ABS-KEY("Incisional Hernia") OR TITLE-ABS-KEY("Incisional Hernias") OR TITLE-ABS-KEY("Postoperative Hernia") OR TITLE-ABS-KEY("Postoperative Hernias") OR TITLE-ABS-KEY("midline hernia") OR TITLE-ABS-KEY("lateral hernia") OR TITLE-ABS-KEY("midline hernias") OR TITLE-ABS-KEY("lateral hernias")

OR

(TITLE-ABS-KEY("Ventral*") OR TITLE-ABS-KEY("umbilical*") OR TITLE-ABS-KEY("incisional*") OR TITLE-ABS-KEY("epigastric*") OR TITLE-ABS-KEY("Spigelian*")) AND (TITLE-ABS-KEY("hernia*"))

AND

TITLE-ABS-KEY("Biosynthetic Mesh") OR TITLE-ABS-KEY("Biosynthetic Meshes") OR TITLE-ABS-KEY("biological Meshes") OR TITLE-ABS-KEY("biological Mesh") OR TITLE-ABS-KEY("synthetic mesh") OR TITLE-ABS-KEY("synthetic meshes") OR TITLE-ABS-KEY("GORE BIO-A") OR TITLE-ABS-KEY("TIGR Matrix") OR TITLE-ABS-KEY("Phasix mesh")


OR

(TITLE-ABS-KEY("Biosynthetic*") OR TITLE-ABS-KEY("biological*") OR TITLE-ABS-KEY("synthetic*") OR TITLE-ABS-KEY("biologic*") OR TITLE-ABS-KEY("Phasix") OR TITLE-ABS-KEY("Bio-a") OR TITLE-ABS-KEY("Tigr") OR TITLE-ABS-KEY("Seri") OR TITLE-ABS-KEY("Reinforcement prosthesis") OR TITLE-ABS-KEY("polyglycolic") OR TITLE-ABS-KEY("trimethylene carbonate") OR TITLE-ABS-KEY("polylactide") OR TITLE-ABS-KEY("poly-4-hydroxybutyrate") OR TITLE-ABS-KEY("P4HB") OR TITLE-ABS-KEY("reabsorbable") OR TITLE-ABS-KEY("absorbable")) AND (TITLE-ABS-KEY("Mesh*"))

**Supplementary file 2**

| *Table 1. Surgical characteristics and outcomes* | | | | | | | | | | | |
| --- | --- | --- | --- | --- | --- | --- | --- | --- | --- | --- | --- |
| **Author (year)** | **Mesh type, n** | **SSO**  **n, (%)** | | | **SSI (deep, superficial) n, (%)** | **Hernia recurrence n, (%)** | **30-day reintervention**  **n, (%)** | **Infected Mesh Removal n (%)** | **Length of hospital stay (days)** | **Technique and Mesh Location** | |
|  |  | *Seroma* | *Hematoma* | *Any* |  |  |  |  |  | **Operative technique** | **Mesh location**  **n, (%)** |
| Bondre et al^21^  (2016) | Synthetic (303) | - | - | - | 54 (17.8) | 41 (13.5) | 33 (10.9) | - | - | - | - |
|  | Biologic (167) | - | - | - | 35 (21.0) | 36 (21.5) | 26 (15.6) | - | - | - | - |
| Brescia et al^22^ (2016) | Synthetic (32) | 5 (15.6) | 3 (9.4) | - | - | 2 (6.3 %) | - | - | 4.3 | - | IP underlay |
|  | Biologic (32) | 3 (9.3) | 2 (6.2) | - | - | 0 | - | - | 4.1 | - |  |
| Buell et al^14^ (2016) | Biosynthetic (31) | - | - | - | 4 (12.9) | 2.01 (6.5) | 2.01 (6.5) | - | 6.7 | Fascial closure | Mesh overlay |
|  | Biologic (42) | - | - | - | (31.0) | 9.99 (23.8) | 6 (14.3) | - | 7.2 | Fascial closure | Mesh overlay |
| Chamieh et al^23^ (2017) | Synthetic (24) | 0 | - | - | 5 (55.6) | - | 0 | - | 7 | CST | Onlay 3 (12.5), RR 21 (87.5) |
|  | Biologic (34) | 6 (33.3) | - | - | 4 (22.2) | - | 3 (8.8) | - | 11.09 | - | IPOM 18 (53), Onlay 6 (17.6), RR 10 (29.4) |
| de Vries et al^24^ (2020) | Synthetic (36) | - | - | - | - | 5 (31.3) | - | - | - | Fascial Closure/CST | Sublay IA, RR, Onlay, Bridging IA sublay |
|  | Biologic (69) | - | - | - | - | 13 (20.6) | - | - | - | Fascial Closure/CST | Sublay IA, RR, Onlay, Bridging IA sublay |
| DeNoto et al^25^ (2013) | Synthetic (268) | 15 (5.6) |  |  | 70 (26.1) | 62 (23.1) | - | 61 (22.8) | 5.5-11.1 | - | - |
|  | Biologic (177) | 7 (3.9) |  |  | 16 (9) | 25 (14.12) | - | 2 (3.6) | 5.3 | - | - |
| El-Gazzaz et al^26^ (2012) | Synthetic (15) | - |  | - | 6 (40) | 6 (40) | 5 (33.3) | 6 (40) | - | - | IP (100) |
|  | Biologic (10) | - | - | - | 5 (50) | 3 (30) | 2 (20) | 3 (30) | - | - | IP (100) |
| Finch et al^27^ (2021) | Biosynthetic (56) | 18 (32.1) | 5 (8.9) | 45 (80.3) | 15 (26.8) | 2 (3.6) | - | 0 | 7 | CST | RR (100) |
| Fischer et al^28^ (2014) | Synthetic (45) | 3.01 (6.7) | (6.7) | 14.98 (33.3) | 5.98 (13.3) | 0 | (8.9) | 1.98 (4.4) | 6.6 | Fascial closure/CST | RR (100) |
|  | Biologic (27) | 1 (3.7) | 0 | 10 (37.0) | 4.99 (18.5) | 3.99 (14.8) | 1 (3.7) | 0.99 (3.7) | 10.2 | Fascial closure/CST | IP underlay (100) |
| Harris et al^8^ (2021) | Synthetic (83) | 10 (12) | 0 | 18 (22) | 28 (34%) | 18 (22) | - | - | 7.5 | CST (anterior) | Onlay 23 (28), sublay-RR 25 (30), underlay 26 (31) |
|  | Biologic (82) | 6 (7) | 4 (5) | 17 (21) | 32 (39%) | 32 (39) | - | - | 8.2 | - | Onlay 14 (17), sublay-RR 31 (38), underlay 25 (30) |
| Koscielny et al^29^ (2018) | Synthetic (24) | 5 (20.8) | 4 (16.7) | 12 (50) | 5 (20,8) | 3 (12.5) | - | - | 12.0 | CST 4 | Retromuscular/sublay 14, Onlay 6, Underlay/IPOM 4 |
|  | Biologic (24) | 7 (29.2) | 6 (25.0) | 19 (79) | 6 (25.0) | 6 (25.0) | - | - | 22.0 | CST 4 | Retromuscular/sublay 14, Onlay 6, Underlay/IPOM 4 |
| López-Cano et al^30^ (2017) | Synthetic (48) | 13 (27.1) | 1 (2.1) | 20 (41.6) | 9 (18.7) | 4 (8.3) | - | - | 11.8 | CST, CT, Others | Onlay 35 (72.9), Sublay 13 (27.1) |
|  | Biologic (14) | 1 (7.1) | 1 (7.1) | 5 (35.7) | 5 (35.7) | 5 (35.7) | - | - | 26.5 | CST, CT, Others | Onlay 10 (71.4), Sublay 4 (28.6) |
| Majumder et al^12^ (2016) | Synthetic (57) | 2 (3.5) | 1 (1.8) | 13 (22.8) | 7 (12.3) | 4 (8.9) | - | 1 (1.8) | 7.7 | CST (89.5) | Retromuscular (98.2), onlay (1.8) |
|  | Biologic (69) | 3 (4.3) | 1 (1.4) | 29 (42) | 22 (31.9) | 15 (26.3) | - | 2 (2.9) | 10.8 | CST (88.4) | Retromuscular (98.6), underlay (1.4) |
| Messa et al^31^ (2019) | Biosynthetic (60) | 6 (6.8) | - | 21 (30) | 6 (8) | 4 (5.7) | 8 (11) | 0 | 4 | Uni./ Bilat. anterior CST,  Uni./ Bilat. posterior CST,  TAR + EOR | Retromuscular 56 (80), onlay 14 (20) |
| Nockolds et al^32^ (2014) | Synthetic (6) | 1 | - | - | 1 | 1 | - | - | - | CST | Onlay 13, sublay 3, both 4, inlay 3 |
|  | Biologic (14) | 4 | - | - | 2 | 2 | - | - | - | CST | Onlay 13, sublay 3, both 4, inlay 3 |
| Olavarria et al^20^ (2020) | Synthetic (43) | 5 (11.6) | 2 (4.7) | - | 4 (9.3) | 5 (13.5) | 3 (8.1) | 0 | 3.0 | Fascial closure | Retromuscular 43 (100) |
|  | Biologic (44) | 5 (11.4) | 3 (6.8) | - | 7 (15.9) | 10 (30.3) | 4 (12.1) | 0 | 3.5 | Fascial closure | Retromuscular 44 (100) |
| Pakula et al^33^ (2020) | Biosynthetic (20) | 2 (10) | - | 4 (20) | 2 (10) | 0 | 0 | - | 5 | TAR, Rives-Stoppa | Retromuscular (100) |
| Plymale et al^36^ (2018) | Biosynthetic (31) | 4 (12.9) | - | 6 (19.4) | 0 | 0 | - | 0 | - | Rives-Stoppa | RR (100) |
| Renard et al^35^ (2020) | Absorbable (57) | 1 (1.8) | 0 | - | 35 (61.4) | 39 (68.4) | 5 (8.8) | - | 12 | - | Intraperitoneal (100) |
|  | Biologic (24) | 1 (4.2) | 1 (4.2) | - | 8 (33.3) | 4 (16.7) | 1 (4.2) | - | 14 | - | IP (100) |
| Rognoni et al^3^ (2020) | Biosynthetic (75) | 5 (6.7) | - | - | 3 (4) | 6 (8) | 4 (5.3) | 1 (1.3) | 11 | Rives–Stoppa, TAR, EOR, IPOM | IP 14 (19),  Onlay 3 (4), RR/preperitoneal 55 (73)  Not reported 3 (4) |
| Rosen et al^6^ (2017) | Biosynthetic (104) | 6 (6) | 1 (1) | 33 (28) | 21 (18) | 13 (14) | 5 | - | 7 | CST | Sublay, IP 10 (10), RR 94 (90) |
| Roth et al^37^ (2021) | Biosynthetic (121) | 8 (6,6) | - | - | 11 (9,3) | 7 (17,9) | 14 (11,6) | - | 5.3 | - | RR without MR 43 (35.5)  RR with MR 45 (37.2)  Onlay without MR 24 (19.8)  Onlay with MR 8 (6.6)  Other 1 (0.8) |
| Sahoo et al^34^ (2017) | Synthetic (380) | 7 (4.0) | 1 (0.6) | 29 (16.7) | 19 (10.9) | - | 7 (4) | - | 6 | Fascial closure | Sublay: RR 203 (58) preperitoneal, 147 (42) |
|  | Biosynthetic (58) | 1 (1.7) | 2 (3.4) | 12 (20.7) | 13 (22.4) | - | 8 (13.8) | - | 6 | - | Sublay: 8 (16) IP, RR 27 (53), preperitoneal 16 (31) |
| van Rooijen et al^10^ (2020) | Biosynthetic (84) | 7 | 2 | 22 (26.2) | 11 | 0 | - | - | - | CST | RR with CST 48 (57.1), RR without CST 35 (41.7), Onlay, with CST 1 (1.2), |
| Vauclair et al^1^ (2021) | Biosynthetic (29) | - | 1 | 9 (31) | - | 3 (10.3) | - | - | 11.2 | CST 2 | IP 10 (34.5), Retromuscular 19 (65.5) |

Supplementary file 3. Bias Publication

Doi plots base on **Seroma** by Subgroup of mesh type.

**Synthetic**

**

**Biologic**

**

**Byosinthetic**

Doi plots base on **SSI** by Subgroup of mesh type.

**Synthetic**

**

**Biologic**

**

**Byosinthetic**

Doi plots base on **Hernia Recurrence** by Subgroup of mesh type.

**Synthetic**** **Biologic** **

**Byosinthetic****

Doi plots base on **Infected Mesh** by Subgroup of mesh type.

**Synthetic**

**

**Biologic**

**

**Byosinthetic**

Doi plots base on ***Reintervention*** by Subgroup of mesh type.

**Synthetic**

**

**Biologic**

**

**Byosinthetic**

**

Figure legend: The vertical line on the horizontal (x) axis represents the effect size (ES) with the lowest absolute z score, dividing the plot into two regions with the same areas. The obtained Luis Furuya Kanamori (LFK) index of xx also suggests major assymmetry.

Supplementary file 4. Subgroup analysis

Table 1. Subgroup analysis (long-term complications)

|  | No. studies | Sample size | Events | Pooled proportion (95% CI) | I^2^ (%) |
| --- | --- | --- | --- | --- | --- |
| *Seroma* | | | | | |
| Synthetic | 9 | 606 | 59 | 11% (6%-17%) | 68.72% |
| Biologic | 11 | 544 | 43 | 9% (5%-13%) | 56.72% |
| Biosynthetic | 8 | 524 | 50 | 9% (4%-15%) | 75.64% |
| *SSI* |  | | | | |
| Synthetic | 10 | 892 | 190 | 20% (15%-25%) | 62.64% |
| Biologic | 13 | 731 | 178 | 27% (17%-38%) | 87.71% |
| Biosynthetic | 9 | 555 | 97 | 15% (6%-27%) | 91.26% |
| *Infected Mesh* |  | | | | |
| Synthetic | 5 | 428 | 70 | 9% (0%-25%) | 92.33% |
| Biologic | 5 | 327 | 8 | 2% (0-7%) | 64.60% |
| Biosynthetic | 4 | 222 | 1 | 0 % (0%-2%) | 0% |
| *Hernia Recurrence* |  | | | | |
| Synthetic | 12 | 940 | 151 | 13% (8%-19%) | 77.59% |
| Biologic | 14 | 792 | 165 | 20% (14%-25%) | 69.53% |
| Biosynthetic | 10 | 668 | 86 | 9% (2%-19%) | 93.32% |
| *Re-intervention* |  | | | | |
| Synthetic | 3 | 361 | 38 | 10% (7%-13%) | 55.82% |
| Biologic | 7 | 348 | 43 | 11% (8%-15%) | 3.38% |
| Biosynthetic | 6 | 393 | 34 | 8% (4%-12%) | 37.47% |
| *Abbreviations: SSI (Surgical Site Infection); IC (Confidence Interval)* | | | | | |

Supplementary file 4. Subgroup analysis

Table 2. Subgroup analysis (risk factor: BMI)

|  | Subgroup: % patient with BMI≥30 | | | | | Subgroup:% patient with BMI<30 | | | | |
| --- | --- | --- | --- | --- | --- | --- | --- | --- | --- | --- |
|  | **No. studies** | **N** | **Proportion** | **(95% CI)** | **I2 (%)** | **No. studies** | **N** | **Proportion** | **(95% CI)** | **I2 (%)** |
| *Seroma* |  |  |  |  |  |  |  |  |  |  |
| Synthetic | 4 | 359 | 6% | 2%-9% | 38% | 3 | 324 | 12% | 2%-21% |  |
| Biologic | 4 | 202 | 5% | 2%-8% | 0 | 3 | 233 | 11% | 0-22% |  |
| Biosynthetic | 3 | 149 | 7% | 1%-14% |  | 4 | 301 | 10% | 2%-17% | 87.34% |
| *SSI* |  |  |  |  |  |  |  |  |  |  |
| Synthetic | 5 | 662 | 17% | 11%-23% | 77.70% | 3 | 307 | 26% | 21%-31% |  |
| Biologic | 6 | 411 | 36% | 21%-51% | 90.92% | 3 | 211 | 23% | 3%-44% |  |
| Biosynthetic | 3 | 149 | 9% | 0%-25% | - | 5 | 332 | 26% | 12%-41% | 91.37% |
| *Infected Mesh* |  |  |  |  |  |  |  |  |  |  |
| Synthetic | 2 | 102 | 3% | 0%-7% | - | 2 | 283 | 23% | 18%-28% |  |
| Biologic | 2 | 96 | 3% | 0%-8% | - | 2 | 187 | 0% | 0%-3% |  |
| Biosynthetic | 2 | 91 | 0% | 0%-2% | - | 1 | 56 | 0% | 0%-6% |  |
| *Hernia Recurrence* |  |  |  |  |  |  |  |  |  |  |
| Synthetic | 4 | 488 | 9% 15 | 2%-19% | 86.49% | 5 | 355 | 20% | 11%-31% | 62.04% |
| Biologic | 6 | 411 | 24% | 17%-31% | 54.85% | 5 | 306 | 14% | 5%-26% | 77.39% |
| Biosynthetic | 3 | 120 | 4% 11 | 0%-13% |  | 5 | 332 | 13% | 0-39% | 96.75% |
| *Reintervention* |  |  |  |  |  |  |  |  |  |  |
| Synthetic | 2 | 477 | 7% | 5%-9% |  | 1 | 15 | 13% | 4%-38% |  |
| Biologic | 2 | 260 | 9% | 3%-16% | 68.83% | 1 | 10 | 20% | 6%-51% |  |
| Biosynthetic | 2 | 118 | 14% | 7%-20% |  | 3 | 192 | 6% | 3%-9% |  |

Table 3. Subgroup analysis (risk factor: smoker)

|  | Subgroup: smoker≥25% | | | | | Subgroup: smoker<25% | | | | |
| --- | --- | --- | --- | --- | --- | --- | --- | --- | --- | --- |
|  | **No. studies** | **Sample Size** | **Proportion** | **(95% CI)** | **I2 (%)** | **No. studies** | **Sample size** | **Proportion** | **(95% CI)** | **I2 (%)** |
| *Seroma* |  |  |  |  |  |  |  |  |  |  |
| Synthetic | 3 | 117 | 17% | 3%-31% | - | 4 | 357 | 6% | 2%-10% | 53.9% |
| Biologic | 3 | 65 | 11% | 0%-24% | - | 4 | 219 | 6% | 3%-9% |  |
| Biosynthetic | 3 | 219 | 8% | 4%-12% | - | 6 | 427 | 7% | 3%-12% | 82.03% |
| *SSI* |  |  |  |  |  |  |  |  |  |  |
| Synthetic | 5 | 435 | 18% | 14-22 | 0.67% | 4 | 357 | 16% | 7%-28% | 85.02% |
| Biologic | 5 | 242 | 23% | 17-30 | 14.82% | 5 | 261 | 38% | 21%-57% | 88.33% |
| Biosynthetic | 3 | 219 | 8% | 3-14 | - | 7 | 458 | 19% | 8%-34% | 92.01% |
| *Infected Mesh* |  |  |  |  |  |  |  |  |  |  |
| Synthetic | 2 | 60 | 10% | 3%-20% |  | 2 | 100 | 0% | 0%-2% |  |
| Biologic | 2 | 37 | 8% | 1%-20% |  | 2 | 113 | 1% | 0%-5% |  |
| Biosynthetic | 2 | 135 | 1% | 0%-3% |  | 2 | 87 | 0% | 0%-2% |  |
| *Hernia Recurrence* |  |  |  |  |  |  |  |  |  |  |
| Synthetic | 5 | 435 | 10% | 3%-22% | 82.23% | 4 | 199 | 15% | 7%-26% | 65.71% |
| Biologic | 5 | 242 | 21% | 16%-27% | 0 | 6 | 324 | 25% | 18%-32% | 45.31% |
| Biosynthetic | 3 | 219 | 4% | 0%-12% |  | 7 | 429 | 13% | 2%-30% | 93.87% |
| *Reintervention* |  |  |  |  |  |  |  |  |  |  |
| Synthetic | 2 | 318 | 11% | 8%-14% |  | 2 | 217 | 4% | 2%-7% |  |
| Biologic | 3 | 204 | 11% | 1%-21% |  | 3 | 110 | 8% | 3%-14% |  |
| Biosynthetic | 1 | 60 | 13% | 7%-24% |  | 5 | 371 | 8% | 5%-12% | 28.75% |

Table 4. Subgroup analysis (design: prospective and retrospective)

|  | Subgroup: Prospective | | | | | Subgroup: Retrospective | | | | |
| --- | --- | --- | --- | --- | --- | --- | --- | --- | --- | --- |
|  | **No. studies** | **N** | **Proportion** | **(95% CI)** | **I2 (%)** | **No. studies** | **N** | **Proportion** | **(95% CI)** | **I2 (%)** |
| *Seroma* |  |  |  |  |  |  |  |  |  |  |
| Synthetic | 2 | 126 | 12% | 6%-18% | - | 9 | 678 | 8% | 3%-14% | 74.47 |
| Biologic | 2 | 126 | 8% | 4%-13% | - | 9 | 418 | 7% | 3%-11% | 47.66 |
| Biosynthetic | 5 | 415 | 7% | 4%-9% | 78.87 | 5 | 251 | 9% | 2%-16% | 85.12 |
| *SSI* |  |  |  |  |  |  |  |  |  |  |
| Synthetic | 2 | 126 | 20% | 13%-26% | - | 10 | 964 | 18% | 13%-23% | 62.10 |
| Biologic | 2 | 126 | 28% | 20%-35% | - | 11 | 605 | 28% | 17%-39% | 90.29 |
| Biosynthetic | 5 | 415 | 8% | 3%-16% | 80.30 | 5 | 282 | 24% | 9%-38% | 90.54 |
| *Infected Mesh* |  |  |  |  |  |  |  |  |  |  |
| Synthetic | 1 | 43 | 0% | 0%-8% | - | 4 | 385 | 14% | 1%-27% | 94.43 |
| Biologic | 1 | 44 | 0% | 0%-8% | - | 4 | 283 | 2% | 1%-5% | 39.14 |
| Biosynthetic | 2 | 106 | 1% | 0%-2% | 0 | 2 | 116 | 0% | 0%-2% | - |
| *Hernia Recurrence* |  |  |  |  |  |  |  |  |  |  |
| Synthetic | 2 | 126 | 17% | 11%-24% | - | 10 | 814 | 12% | 6%-19% | 80.56 |
| Biologic | 2 | 126 | 32% | 24%-40% | - | 12 | 666 | 17% | 12%-23% | 58.14 |
| Biosynthetic | 5 | 444 | 6% | 1%-13% | 84.02 | 5 | 224 | 10% | 0%-39% | 96.22 |
| *Reintervention* |  |  |  |  |  |  |  |  |  |  |
| Synthetic | 1 | 43 | 7% | 2%-19% | - | 4 | 516 | 6% | 1%-12% | 74.51 |
| Biologic | 1 | 44 | 9% | 4%-21% | - | 6 | 304 | 11% | 5%-15% | 51.48 |
| Biosynthetic | 2 | 225 | 8% | 4%-10% | - | 5 | 226 | 6% | 1%-14% | 66.99 |

Table 5. Subgroup analysis (risk of bias)

|  | Low risk of bias | | | | | Medium risk of bias | | | | |
| --- | --- | --- | --- | --- | --- | --- | --- | --- | --- | --- |
|  | **No. studies** | **N** | **Proportion** | **(95% CI)** | **I2 (%)** | **No. studies** | **N** | **Proportion** | **(95% CI)** | **I2 (%)** |
| *Seroma* |  |  |  |  |  |  |  |  |  |  |
| Synthetic | 8 | 506 | 10% | 6%-15% | 69.69% | 3 | 298 | 6% | 3%-8% | - |
| Biologic | 8 | 316 | 7% | 4%-10% | 23% | 3 | 228 | 13% | 0%-25% | - |
| Biosynthetic | 2 | 115 | 2% | 0%-4% | - | 8 | 551 | 10% | 6%-14% | 60% |
| *SSI* |  |  |  |  |  |  |  |  |  |  |
| Synthetic | 10 | 792 | 17% | 12%-22% | 67.20% | 3 | 298 | 25% | 20%-30% |  |
| Biologic | 10 | 503 | 34% | 23%-45% | 85.91% | 3 | 228 | 10% | 6%-13% |  |
| Biosynthetic | 3 | 146 | 32% | 4%-60% | - | 8 | 551 | 12% | 7%-18% | 73.46 |
| *Infected Mesh* |  |  |  |  |  |  |  |  |  |  |
| Synthetic | 4 | 160 | 6% | 0%-19% | 83.49% | 1 | 268 | 23% | 18%-28% | - |
| Biologic | 4 | 150 | 3% | 0%-13% | 68.56% | 1 | 177 | 1% | 0%-14% | - |
| Biosynthetic | - | - | - | - | - | 4 | 222 | 0% | 0-2% | 0% |
| *Hernia Recurrence* |  |  |  |  |  |  |  |  |  |  |
| Synthetic | 10 | 666 | 12% | 7%-18% | 73.81% | 2 | 580 | 23% | 18%-28% | - |
| Biologic | 12 | 598 | 21% | 15%-28% | 69.23% | 2 | 194 | 14% | 9%-19% | - |
| Biosynthetic | 2 | 88 | 44% | 34%-55% | - | 9 | 274 | 5% | 2%-10% | 76.75% |
| *Reintervention* |  |  |  |  |  |  |  |  |  |  |
| Synthetic | 4 | 535 | 8% | 3%-12% | 67.53% | 1 | 24 | 0% | 0%-14% | - |
| Biologic | 6 | 314 | 10% | 5%-15% | 51.41% | 1 | 34 | 9% | 3%-23% | - |
| Biosynthetic | 0 | 146 | 10% | 5%-14% | - | 4 | 305 | 7% | 3%-14% | 62.13% |
